# Cytopathologic, Histopathologic, and Immunohistochemical Features of Intrahepatic Clear Cell Bile Duct Adenoma: A Case Report and Review of the Literature

**DOI:** 10.1155/2014/874826

**Published:** 2014-05-19

**Authors:** William W. Wu, Mai Gu, Di Lu

**Affiliations:** Department of Pathology and Laboratory Medicine, University of California Irvine Medical Center, 101 The City Drive, Irvine, Orange, CA 92868, USA

## Abstract

Intrahepatic clear cell bile duct adenoma is extremely rare, with only 3 previous cases reported in the literature. The cause of cytoplasmic clearing in clear cell bile duct adenoma has not been previously investigated. Distinguishing clear cell bile duct adenoma from other clear cell tumors, particularly clear cell cholangiocarcinoma, can be challenging. Previous studies have shown loss of CD10 expression and focal CD56 expression in cholangiocarcinoma. Expressions of CD10 and CD56 have not been previously studied in clear cell bile duct adenoma. A 37-year-old morbidly obese woman was diagnosed with a 2.8 cm intrahepatic clear cell bile duct adenoma following segmental hepatic resection. Histochemical analysis of the tumor suggested the cause of cytoplasmic clearing in the neoplastic cells to be mucin and not glycogen or lipid. On immunohistochemical staining, the neoplastic cells demonstrated staining for CK7, CA 19-9, polyclonal CEA, CD10 (apical), CD56 (focal), and vimentin. Ki-67 highlighted less than 2% of tumor cell nuclei. This is the first report to study the etiology of cell clearing in clear cell bile duct adenoma. Expression of CD10 in clear cell bile duct adenoma may help distinguish clear cell bile duct adenoma from clear cell cholangiocarcinoma.

## 1. Introduction

Intrahepatic bile duct adenomas, also known as peribiliary gland hamartomas, are uncommon tumors that are usually found incidentally during surgery or at autopsy. The incidence of bile duct adenoma has been estimated at 1.3% [1]; however, the true incidence of this entity is unknown.

The clear cell variant of intrahepatic bile duct adenoma is an extremely rare entity. To date, only 3 cases have been reported in the literature [2]. In this report, a case of intrahepatic clear cell bile duct adenoma with background of marked hepatic steatosis is presented. The radiologic, cytopathologic, histopathologic, and immunohistochemistochemical findings are presented, with review of literature and discussion of mechanism of clear cell change and features that may help distinguish clear cell bile duct adenoma from clear cell cholangiocarcinoma.

## 2. Case Report

A 37-year-old morbidly obese diabetic woman with no prior history of malignancy was found to have elevated liver function tests (LFTs), elevated CA 19-9, and a liver mass at an outside hospital. The patient was referred to our facility for further management. Her family history was significant for pancreatic cancer in the maternal grandmother. Eight years prior, the patient had elevated liver function tests on routine physical examination. Subsequent workup at that time revealed a liver lesion, and biopsy of the lesion showed liver steatosis but was negative for malignancy. No additional workup was performed.

**Figure 1:**
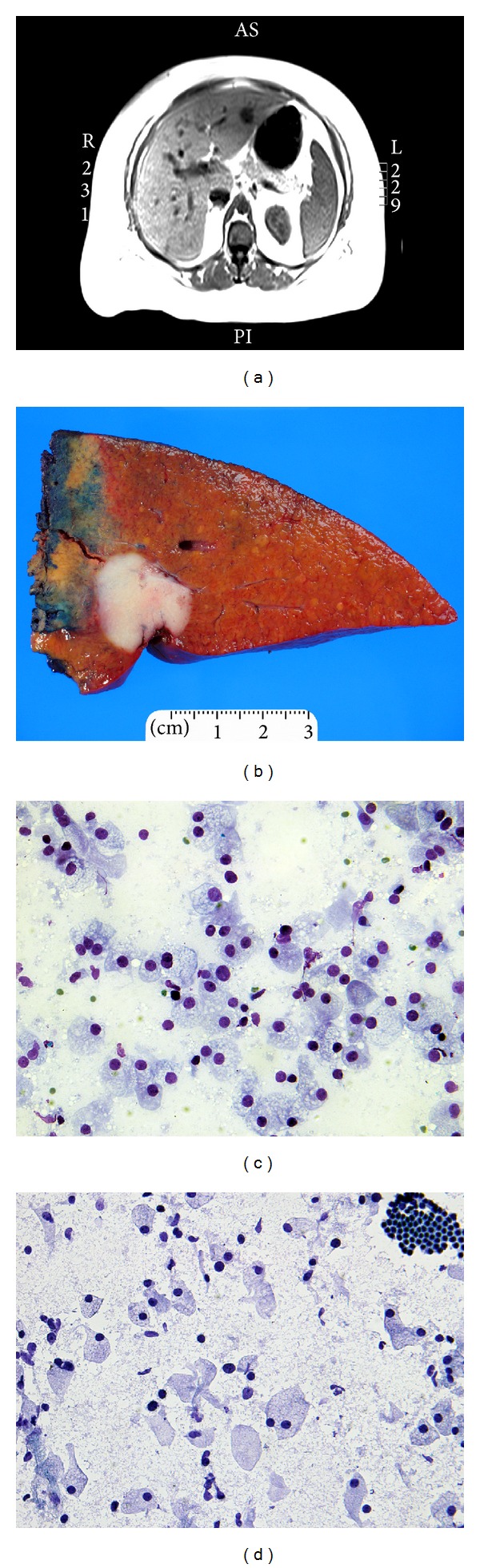
Radiologic, macroscopic, and cytologic features of intrahepatic clear cell bile duct adenoma. (a) Axial T1-weighted MRI scan of the abdomen demonstrating a hypointense lesion in the left lateral segment of the liver. (b) Segmental hepatectomy specimen showing a tan-white well circumscribed subcapsular tumor measuring 2.8 × 2.2 × 2.0 cm. (c-d) Diff-Quick (c) and ultrafast Papanicolaou (d) stained cytologic smears of tumor cells (c-d, ×400).

Imaging study revealed a lesion in the left lateral lobe of the liver, suspicious for metastatic carcinoma (Figure 1(a)). In addition, endoscopic ultrasound showed heterogenous echogenicity with lobulation in the entire pancreas, compatible with fatty infiltration of the pancreas. No discrete pancreatic mass was identified. A fine needle aspiration of the pancreatic head showed benign acinar and glandular cells and was negative for malignancy. No additional workup was performed on the aspirated material from the pancreas. A core biopsy of the liver lesion showed a clear cell tumor. A segmental liver resection to remove the tumor was performed. Following surgery, the patient developed a seroma at the site of hepatic lobectomy that has since spontaneously resolved. At her latest follow-up visit, the patient's LFTs were mildly elevated with AST of 48 IU/L, ALT of 40 IU/L, and an alkaline phosphatase of 120 IU/L. Her tumor markers were unremarkable, with CA 19-9 of 15 U/mL, CA-125 of 5 U/mL, and CEA of 1.3 ng/mL. The patient is alive 19 months after surgery with no recurrence of tumor.

## 3. Results

### 3.1. Pathologic Evaluation

Gross examination of the segmental liver resection specimen revealed a tan-white well circumscribed subcapsular tumor measuring 2.8 × 2.2 × 2 cm with steatosis in the adjacent liver parenchyma (Figure 1(b)). The resected margin was grossly and microscopically negative for tumor. Cytologic smear preparations were obtained from the liver mass. A representative portion of fresh tumor was frozen, sectioned, and submitted for Oil Red O staining. The remainder of the tumor was entirely submitted, fixed in 10% buffered formalin, embedded in paraffin, and stained with hematoxylin and eosin (H&E). Trichrome, reticulin, periodic acid-schiff (PAS), PAS with diastase predigestion, iron, mucicarmine, Alcian blue, and colloidal iron histochemical stains were performed. Immunohistochemical stains for cytokeratin 7 (CK7), cytokeratin 20 (CK20), epithelial membrane antigen (EMA), polyclonal carcinoembryonic antigen (CEA), CA 19-9, CD10, CD56, renal cell carcinoma marker (RCC), hepatocellular carcinoma marker (HCC), vimentin, p53, Ki-67, TTF-1, estrogen receptor (ER), progesterone receptor (PR), and chromogranin A were all performed using standard avidin-peroxidase techniques.

### 3.2. Cytopathologic Features

Direct smears of scrape preparation of the hepatic mass demonstrated cells with abundant foamy cytoplasm and eccentrically located hyperchromatic round-to-ovoid nuclei with minimal cytologic atypia (Figures 1(c)-1(d)). The tumor cells were arranged in small groups or individual cells. Occasional inflammatory cells (lymphocytes and neutrophils) in the background were identified. Tumor cell necrosis, mitosis, or significant cytologic atypia were absent.

### 3.3. Histopathologic Features

**Figure 2:**
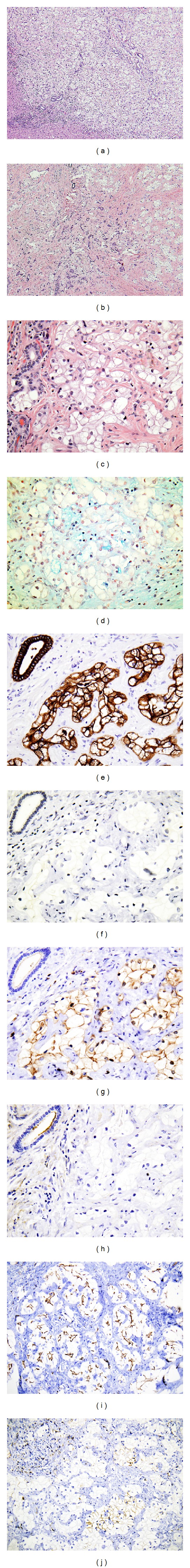
Microscopic and immunohistochemical features of intrahepatic clear cell bile duct adenoma. (a) Hematoxylin-eosin stained low power view showing tumor composed almost exclusively of clear cells with entrapped bile ducts and surrounding liver parenchyma. (b) Stromal fibrosis and partial replacement of normal bile duct cells by clear cells. (c) The tumor cells have abundant clear cytoplasm and round to oval hyperchromatic nuclei with inconspicuous nucleoli. (d) The clear cells are focally positive for Alcian blue staining. (e–h) Immunohistochemical staining of clear cells demonstrates activity for CK7 (e) and polyclonal CEA (g) but negative staining for CK20 (f) and EMA (h). (g-h) Clear tumor cells demonstrated continuous apical staining for CD10 (g) and focal membraneous staining for CD56 (h) (a-b, ×100; c–h, ×400; i-j, ×200).

Histologic sections of the hepatic tumor showed neoplastic cells with abundant clear cytoplasm arranged in tubules and small nests (Figures 2(a)–2(c)). The tumor was almost exclusively composed of clear cells (>99%). Nuclei of the neoplastic cells were round, ovoid, and hyperchromatic. The tumor was well circumscribed with focal invasion into the surrounding liver parenchyma. Areas of mild to moderate stromal fibrosis were seen within the tumor (Figure 2(b)). Partial replacement of entrapped bile ducts by clear cells was identified focally (Figures 2(b) and 2(c)). Dilated bile ducts with lumens containing bile-stained secretions were not identified. No nuclear atypia, mitoses, or tumor necrosis was seen within the tumor. The surrounding liver parenchyma showed marked steatosis (80%) with moderate portal fibrosis and focal bridging. Mild portal chronic inflammation composed of predominantly lymphocytes was identified.

Immunohistochemical staining of neoplastic clear cells showed strong and diffuse staining with CK7 (Figure 2(e)) and CA 19-9, cytoplasmic staining for polyclonal CEA (Figure 2(g)), and membranous staining for vimentin. The tumor cells displayed continuous apical staining with CD10 (Figure 2(i)) and focal membranous staining with CD56 (Figure 2(j)). Weak nuclear staining for p53 was seen in greater than 50% of tumor cell nuclei. The Ki-67 proliferation index in the tumor was estimated at less than 2%. The tumor cells were negative for CK20 and EMA (Figures 2(f) and 2(h)), as well as for HCC marker, RCC marker, TTF-1, ER, PR, CA-125, and chromogranin A.

PAS staining highlighted the basement membrane surrounding tumor cell nests and revealed intracytoplasmic granules within tumor cells that were resistant to diastase digestion. The tumor cells demonstrated staining for Alcian blue (Figure 2(d)) and weak staining for mucicarmine. Oil Red O staining highlighted lipid globules within hepatocytes in the surrounding liver parenchyma but was negative within tumor cells. Trichrome highlighted portal fibrosis and focal bridging in the liver parenchyma. Iron staining revealed no evidence of hemosiderosis. Reticulin staining was not decreased and showed no increase in thickness of hepatic plates.

Although focal microinvasion was identified, the subcapsular location of the tumor, its predominantly well circumscribed border, and the lack of cytologic atypia, mitoses, and necrosis favored a diagnosis of intrahepatic clear cell bile duct adenoma over clear cell cholangiocarcinoma.

## 4. Discussion

Bile duct adenomas, also known as peribiliary gland hamartomas, are uncommon tumors that are usually found incidentally during surgery or at autopsy. The incidence of bile duct adenoma has been estimated at 1.3% [1]; however the true incidence of this entity is unknown. In a study involving 2,125 postmortem examinations, Cho et al. [3] identified only 13 cases of bile duct adenoma. Craig et al. [4] identified only 5 bile duct adenomas in a study of 50,000 autopsies. In the largest series of bile duct adenomas, Allaire et al. [5] found 152 cases between 1943 and 1986. Of these 152 bile duct adenomas, nearly 68% (103 cases) were asymptomatic and discovered incidentally during abdominal surgery, while the remaining cases were found at autopsy (49 cases). Intrahepatic bile duct adenomas are usually subscapsular, ranging in size from 0.1 to 2 cm (mean: 0.6 cm), and mostly affect individuals between 20 and 70 years of age (mean: 55 years) with no significant difference in sex distribution [5]. Bile duct adenomas are usually solitary; however they can occur as multiple nodules [3, 6].

Grossly, bile duct adenomas appear as well-demarcated, tan-white, firm subcapsular nodules. Microscopically, bile duct adenomas are composed of small noncystic tubules and acini surrounded by variably fibrous and inflamed stroma. The epithelial cells lining the tubules are usually low-columnar to cuboidal and are cytologically benign.

The pathogenesis of bile duct adenomas is unclear. It has been proposed that bile duct adenomas are formed by reactive processes to focal bile duct injury caused by trauma or inflammation [5]. Bhathal et al. [7] showed that the phenotype of bile duct adenomas is similar to that of peribiliary glands and have proposed that bile duct adenomas be referred to as peribiliary gland hamartomas. Subsequent work has shown that bile duct adenomas are phenotypically similar to inflamed peribiliary glands, suggesting that bile duct adenomas may indeed form as a response to injury [8].

Clear cell variants of intrahepatic bile duct adenoma are exceedingly rare. Only three clear cell bile duct adenomas have been reported previously in the literature [2]. As such, experience with this entity is very limited. In their report, Albores-Saavedra et al. [2] described intrahepatic clear cell bile duct adenomas as small, subcapsular tumors ranging in size from 0.8 cm to 1.1 cm. Microscopically, the tumors are composed of clear cells with minimal cytologic atypia with focal infiltration into the adjacent liver parenchyma. By immunohistochemistry, clear cell bile duct adenomas are positive for CK7, CEA, and EMA and are negative for CK20, HepPar-1, chromogranin A, prostate specific antigen (PSA), and vimentin [2]. The Ki-67 proliferation index of clear cell bile duct adenomas is usually less than 10%.

At 2.8 cm, the clear cell bile duct adenoma described in this case is the largest to be reported to date. Microscopically, the tumor displayed features nearly identical to previously reported clear cell bile duct adenomas [2]. Interestingly, the tumor in this case showed negative staining for EMA (Figure 2(h)) and membrane staining for vimentin. This differs from the clear cell bile duct adenomas described by Albores-Saavedra et al. [2], which all showed positive EMA staining and negative vimentin staining. The significance of the differences in EMA and vimentin staining is unclear due to the small number of cases and may require further study.

The etiology of the cytoplasmic clearing in clear cell bile duct adenomas has not been previously studied. Clearing of cytoplasm may be due to intracellular accumulation of glycogen, lipid or mucin. The degree of steatosis (80%) in the liver of our patient suggested that lipid may be responsible for the cytoplasmic clearing of tumor cells. However, negative staining of tumor cells with Oil Red O did not support this hypothesis. Identification of PAS-positive material in tumor cells suggested that the cytoplasmic clearing may be due to glycogen or mucin. The diastase-resistant nature of the intracytoplasmic material within tumor cells, in conjunction with positive Alcian blue and mucicarmine staining, demonstrated that mucin is most likely responsible for the cytoplasmic clearing of the tumor cells in this case.

The differential diagnosis for clear cell bile duct adenoma includes bile duct hamartoma (von Meyenburg complex), clear cell cholangiocarcinoma, clear cell hepatocellular carcinoma, and metastatic clear cell carcinomas. Bile duct hamartomas, also known as von Meyenburg complexes, can be differentiated from bile duct adenomas by the presence of dilated ducts filled with bile-stained material, a feature that should not be present in bile duct adenoma.

The negative staining of tumor cells with HCC marker helps rule out clear cell hepatocellular carcinoma in this case. Negative staining of tumor cells with RCC marker, TTF-1, CA-125, ER, and PR help rule out metastatic clear cell carcinomas originating from the kidney, lung, thyroid, ovary, and breast. Negative chromogranin A staining also makes a neuroendocrine tumor with clear cell features less likely in this case. Identification of areas in the tumor with bile ducts partially replaced by clear cells, in conjunction with positive CA 19-9 staining, strongly favors a biliary origin of the tumor.

Differentiating intrahepatic clear cell bile duct adenomas from clear cell cholangiocarcinomas can be diagnostically challenging. Only 9 cases of intrahepatic clear cell cholangiocarcinoma have been reported to date [2, 9–13]. Clear cell cholangiocarcinomas appear to affect men more than women (M : F ratio: 2 : 1), while clear cell bile duct adenomas have no gender predilection (M : F ratio: 1 : 1) (Table 1). There are also no significant differences in patient ages or tumor sizes between clear cell bile duct adenomas and clear cell cholangiocarcinomas (Table 1). Clear cell bile duct adenomas are subcapsular and have well demarcated borders, while clear cell cholangiocarcinomas can be subcapsular or deep and may have irregular/infiltrative borders. Clear cell bile duct adenomas should have minimal or no cytologic atypia, whereas the cells in clear cell cholangiocarcinoma may display more cellular atypia and pleomorphism. Mitoses and areas with necrosis should be absent in clear cell bile duct adenomas but may be present in clear cell cholangiocarcinomas.

By immunohistochemistry, both clear cell bile duct adenomas and clear cell cholangiocarcinomas show positivity for CK7 and negativity for CK20, with some clear cell cholangiocarcinomas demonstrating decreased expression of CK7 [2, 12] (Table 1). No significant differences were identified in the staining patterns for CK20, p53, vimentin, chromogranin A, HCC marker, RCC marker, ER, PR, and TTF-1 between intrahepatic clear cell bile duct adenomas and intrahepatic clear cell cholangiocarcinomas. Although the Ki-67 proliferation index for bile duct adenomas is generally considered to be less than 10%, bile duct adenomas may exhibit Ki-67 indices exceeding 10% [2], while some clear cell cholangiocarcinomas may have Ki-67 indices of less than 10% [12, 13]. Ki-67 alone is insufficient for distinguishing clear cell bile duct adenoma from clear cell cholangiocarcinoma.

A number of reports have shown loss of CD10 expression in malignant extrahepatic bile duct lesions with preservation of CD10 expression in benign lesions [14–22]. Loss of CD10 has been previously reported in intrahepatic clear cell cholangiocarcinomas [12, 13]. Previously reported cases of intrahepatic clear cell bile duct adenoma were not evaluated with CD10 [2]. Interestingly, the tumor in our case demonstrated continuous apical staining with CD10 (Figure 2(i)). Preservation of CD10 expression suggests that the tumor in our case is a benign adenoma rather than an invasive carcinoma. CD10 staining may be useful in distinguishing intrahepatic clear cell bile duct adenoma from intrahepatic clear cell cholangiocarcinoma.

Previous reports have shown focal staining of intrahepatic clear cell cholangiocarcinomas with CD56 [12, 13]. It has been suggested that reactivity for CD56 may be a novel feature of clear cell intrahepatic cholangiocarcinoma. Previous clear cell intrahepatic bile duct adenomas were not stained with CD56 [2]. The tumor in our case showed focal membranous reactivity to CD56 (Figure 2(j)). CD56 reactivity may be related to clear cell change, as previously proposed by Haas et al. [12], and may be seen in both clear cell bile duct adenoma and clear cell cholangiocarcinoma.

The prognosis of clear cell bile duct adenoma is favorable. All the four patients with intrahepatic clear cell bile duct adenoma were alive at the time of report with no evidence of recurrence. The prognosis of intrahepatic clear cell cholangiocarcinoma is relatively good, especially when compared to conventional cholangiocarcinoma. Of the reported cases of intrahepatic clear cell cholangiocarcinoma with available follow-up information, only 2 patients died of their disease at the time of report [2, 10–13, 23]. Due to the extremely limited experience with clear cell bile duct adenomas, careful follow-up is recommended.

In summary, intrahepatic clear cell bile duct adenomas are extremely rare tumors of the liver. We describe the largest intrahepatic clear cell bile duct adenoma to date, originally thought to be a metastatic clear cell carcinoma. Recognition of this entity is important in distinguishing it from other clear cell tumors of the liver, particularly clear cell cholangiocarcinoma.

## Figures and Tables

**Table 1:** Clinicopathologic and immunohistochemical characteristics of reported cases of intrahepatic clear cell bile duct adenoma and clear cell cholangiocarcinoma.

Case number	Reference	Age (y)/ gender	Size (cm)	Follow-up	Immunohistochemistry									
					CK7	CK20	EMA	pCEA	CD10	CD56	Ca 19-9	Vimentin	Ki-67	p53
Intrahepatic clear cell bile duct adenoma														
1	Albores-Saavedra et al. (2001) [2]	50/M	0.8*	Alive, 2 years	+	−	+	+ (cytoplasmic)	nd	nd	nd	−	<10% in 2 of 3 cases	+ (>50%)
2	Albores-Saavedra et al. (2001) [2]	63/M	0.9	Alive, 2 months	+	−	+	+ (cytoplasmic)	nd	nd	nd	−		+ (>50%)
3	Albores-Saavedra et al. (2001) [2]	25/F	1.1	Alive, 18 years	+	−	+	+ (cytoplasmic)	nd	nd	nd	−		+ (>50%)
4	Current case	37/F	2.8	Alive, 19 months	+	−	−	+	+	+ (focal)	+	+ (membrane)	<2%	+ (>50%)

Intrahepatic clear cell cholangiocarcinoma														
1	Logani and Adsay (1998) [9]	64/F	12.0	nd	nd	nd	nd	nd	nd	nd	nd	−	nd	nd
2	Adamek et al. (1998) [10]	62/M	5.0	Died, 14 months	+	nd	+	nd	nd	nd	nd	nd	nd	nd
3	Tihan et al. (1998) [11]	72/M	15.0	Alive, 30 months	+	−	+ (focal)	−	nd	nd	nd	−	nd	nd
4	Falta et al. (1999) [23]	50/M	1.5	Alive, 12 months	+	−	+	−	nd	nd	nd	+ (focal)	nd	nd
5	Albores-Saavedra et al. (2001) [2]	64/M	6.0	Alive, 3 years	+ (>50%)	−	nd	+ (>50%)	nd	nd	nd	nd	>50%	+ (>50%)
6	Haas et al. (2007) [12]	51/M	9.0	Died, 3 years	+	−	−	−	−	+ (20%)	−	+ (20%)	<5%	nd
7	Haas et al. (2007) [12]	60/F	4.5	Alive, 1 year	+ (30%)	−	+ (50%)	−	−	+ (10–20%)	−	−	<2%	nd
8	Haas et al. (2007) [12]	58/F	2.3	Alive	+ (10%)	−	−	−	−	+ (10–60%)	−	+ (20%)	5%	nd
9	Toriyama et al. (2010) [13]	56/M	2.2	Alive, 7 months	+	−	+	−	−	+ (focal)	+ (focal)	+	7.5%	+ (focal)

*2 tumors, each measuring 0.8 cm; +: positive; −: negative; nd: no data.

## References

[1] Edmondson HA (1958). *Tumors of the Liver and Intrahepatic Bile Duct*.

[2] Albores-Saavedra J, Hoang MP, Murakata LA, Sinkre P, Yaziji H (2001). Atypical bile duct adenoma, clear cell type: a previously undescribed tumor of the liver. *The American Journal of Surgical Pathology*.

[3] Cho C, Rullis I, Rogers LS (1978). Bile duct adenomas as liver nodules. *Archives of Surgery*.

[4] Craig JR, Peters RL, Edmondson HA (1989). *Tumors of the Liver and Intrahepatic Bile Ducts*.

[5] Allaire GS, Rabin L, Ishak KG, Sesterhenn IA (1988). Bile duct adenoma: a study of 152 cases. *The American Journal of Surgical Pathology*.

[6] Levin SE, Dail DH, Saik RP (1975). Bile duct adenomatosis of the liver: a misleading finding on surgical exploration of the abdomen. *The American Surgeon*.

[7] Bhathal PS, Hughes NR, Goodman ZD (1996). The so-called bile duct adenoma is a peribiliary gland hamartoma. *The American Journal of Surgical Pathology*.

[8] Hughes NR, Goodman ZD, Bhathal PS (2010). An immunohistochemical profile of the so-called bile duct adenoma: clues to pathogenesis. *The American Journal of Surgical Pathology*.

[9] Logani S, Adsay V (1998). Clear cell cholangiocarcinoma of the liver is a morphologically distinctive entity. *Human Pathology*.

[10] Adamek HE, Spiethoff A, Kaufmann V, Jakobs R, Riemann JF (1998). Primary clear cell carcinoma of noncirrhotic liver: immunohistochemical discrimination of hepatocellular and cholangiocellular origin. *Digestive Diseases and Sciences*.

[11] Tihan T, Blumgart L, Klimstra DS (1998). Clear cell papillary carcinoma of the liver: an unusual variant of peripheral cholangiocarcinoma. *Human Pathology*.

[12] Haas S, Gütgemann I, Wolff M, Fischer H-P (2007). Intrahepatic clear cell cholangiocarcinoma: immunohistochemical aspects in a very rare type of cholangiocarcinoma. *The American Journal of Surgical Pathology*.

[13] Toriyama E, Nanashima A, Hayashi H (2010). A case of intrahepatic clear cell cholangiocarcinoma. *World Journal of Gastroenterology*.

[14] Chu P, Arber DA (2000). Paraffin-section detection of CD10 in 505 nonhematopoietic neoplasms: frequent expression in renal cell carcinoma and endometrial stromal sarcoma. *The American Journal of Clinical Pathology*.

[15] Xiao S, Wang HL, Hart J, Fleming D, Beard MR (2001). cDNA arrays and immunohistochemistry identification of CD10/CALLA expression in hepatocellular carcinoma. *The American Journal of Pathology*.

[16] Lau SK, Prakash S, Geller SA, Alsabeh R (2002). Comparative immunohistochemical profile of hepatocellular carcinoma, cholangiocarcinoma, and metastatic adenocarcinoma. *Human Pathology*.

[17] Morrison C, Marsh W, Frankel WL (2002). A comparison of CD10 to pCEA, MOC-31, and hepatocyte for the distinction of malignant tumors in the liver. *Modern Pathology*.

[18] Shousha S, Gadir F, Peston D, Bansi D, Thillainaygam AV, Murray-Lyon IM (2004). CD10 immunostaining of bile canaliculi in liver biopsies: change of staining pattern with the development of cirrhosis. *Histopathology*.

[19] Röcken C, Licht J, Roessner A, Carl-McGrath S (2005). Canalicular immunostaining of aminopeptidase N (CD13) as a diagnostic marker for hepatocellular carcinoma. *Journal of Clinical Pathology*.

[20] Komuta M, Spee B, Borght SV (2008). Clinicopathological study on cholangiolocellular carcinoma suggesting hepatic progenitor cell origin. *Hepatology*.

[21] Nishihara Y, Aishima S, Hayashi A (2009). CD10+ fibroblasts are more involved in the progression of hilar/extrahepatic cholangiocarcinoma than of peripheral intrahepatic cholangiocarcinoma. *Histopathology*.

[22] Tretiakova M, Antic T, Westerhoff M (2012). Diagnostic utility of CD10 in benign and malignant extrahepatic bile duct lesions. *The American Journal of Surgical Pathology*.

[23] Falta EM, Rubin AD, Harris JA (1999). Peripheral clear cell cholangiocarcinoma: a rare histologic variant. *The American Surgeon*.

